# Neural Oscillations Carry Speech Rhythm through to Comprehension

**DOI:** 10.3389/fpsyg.2012.00320

**Published:** 2012-09-06

**Authors:** Jonathan E. Peelle, Matthew H. Davis

**Affiliations:** ^1^Center for Cognitive Neuroscience and Department of Neurology, University of PennsylvaniaPhiladelphia, PA, USA; ^2^Medical Research Council Cognition and Brain Sciences UnitCambridge, UK

**Keywords:** intelligibility, language, oscillations, phase locking, speech comprehension, speech rate, theta

## Abstract

A key feature of speech is the quasi-regular rhythmic information contained in its slow amplitude modulations. In this article we review the information conveyed by speech rhythm, and the role of ongoing brain oscillations in listeners’ processing of this content. Our starting point is the fact that speech is inherently temporal, and that rhythmic information conveyed by the amplitude envelope contains important markers for place and manner of articulation, segmental information, and speech rate. Behavioral studies demonstrate that amplitude envelope information is relied upon by listeners and plays a key role in speech intelligibility. Extending behavioral findings, data from neuroimaging – particularly electroencephalography (EEG) and magnetoencephalography (MEG) – point to phase locking by ongoing cortical oscillations to low-frequency information (~4–8 Hz) in the speech envelope. This phase modulation effectively encodes a prediction of when important events (such as stressed syllables) are likely to occur, and acts to increase sensitivity to these relevant acoustic cues. We suggest a framework through which such neural entrainment to speech rhythm can explain effects of speech rate on word and segment perception (i.e., that the perception of phonemes and words in connected speech is influenced by preceding speech rate). Neuroanatomically, acoustic amplitude modulations are processed largely bilaterally in auditory cortex, with intelligible speech resulting in differential recruitment of left-hemisphere regions. Notable among these is lateral anterior temporal cortex, which we propose functions in a domain-general fashion to support ongoing memory and integration of meaningful input. Together, the reviewed evidence suggests that low-frequency oscillations in the acoustic speech signal form the foundation of a rhythmic hierarchy supporting spoken language, mirrored by phase-locked oscillations in the human brain.

Listening to speakers of different languages – whether in a cafe, on the television, or over internet radio – quickly reveals rhythmic characteristics which can distinguish many of the world’s languages. These perceptually salient differences include the three-way distinction between the staccato, rapid rhythm of languages such as Japanese, the regular “machine gun” rhythm of languages such as Italian, and the “Morse code” alternations of strong and weak syllables in languages such as Dutch or German (Pike, 1945; Abercrombie, 1967). However, regular timing is far more apparent in the ear of the listener than in the acoustic characteristics of speech: the differences among classes of language prove strikingly elusive when we measure the duration of critical elements of the speech signal (Lehiste, 1977; Dauer, 1983). What function, then, does perceptually salient rhythm play in the comprehension of connected speech? How does the brain make use of rhythmic structure in identifying the acoustic and linguistic units (segments, syllables, and words) that comprise the speech signal and support the computation of meaning?

Here we review the contribution of low-frequency acoustic information in connected speech – that is, speech rhythm – to the comprehension process. We also consider the role of low-frequency neural oscillations in processing ongoing sensory stimuli such as speech, and propose that a complementary relationship between speech rhythm and periodic oscillations in the brain allows the two to come together in supporting spoken language comprehension.

In laying out this proposal, we begin by exploring the temporal characteristics of the speech signal, with a particular focus on information conveyed in amplitude modulations that are linked to the production of spoken syllables. Although it is certainly not true that all aspects of speech processing can be explained with reference to slow amplitude modulations in speech, these are both a salient aspect of the acoustic speech signal and critical for intelligibility, and thus a fundamental part of speech comprehension. We then discuss mechanisms in the brain that support the processing of speech information, with an emphasis on neural responses that are coordinated with the slow fluctuations in the acoustic signal of incoming speech. Finally, we propose an account of how neural processes that respond to these slow amplitude fluctuations operate in the context of a broader neuroanatomical model of spoken language comprehension.

## The Temporal Characteristics of Speech that Contribute to Comprehension

Because speech unfolds over time, both perception and comprehension of spoken language rely on being able to integrate current acoustic information with past input, and on assessing the direction and rate of change in acoustic information. A consequence of this temporal dependence in speech processing is that listeners must process the incoming acoustic signal in near real-time, as they are rarely provided with an opportunity to hear the same speech signal again. In addition, the rate at which information is processed during speech comprehension is determined not by the needs of the listener, but at a rate determined by the speaker (in contrast to reading comprehension, in which the reader determines the rate at which the eyes scan the page). These temporal constraints put considerable pressure on listeners to process speech as efficiently as possible, and therefore in a way that is optimally tuned to the rate of the incoming speech signal. As we will see, these temporal constraints have important implications for the operation of neural systems involved in speech processing, and in particular how these systems must combine their own temporal preferences with the temporal structure of incoming speech. An important first step in this investigation is to identify the timescale at which various types of speech cues are produced and comprehended.

### Low-frequency amplitude modulations in speech

**Figure 1:**
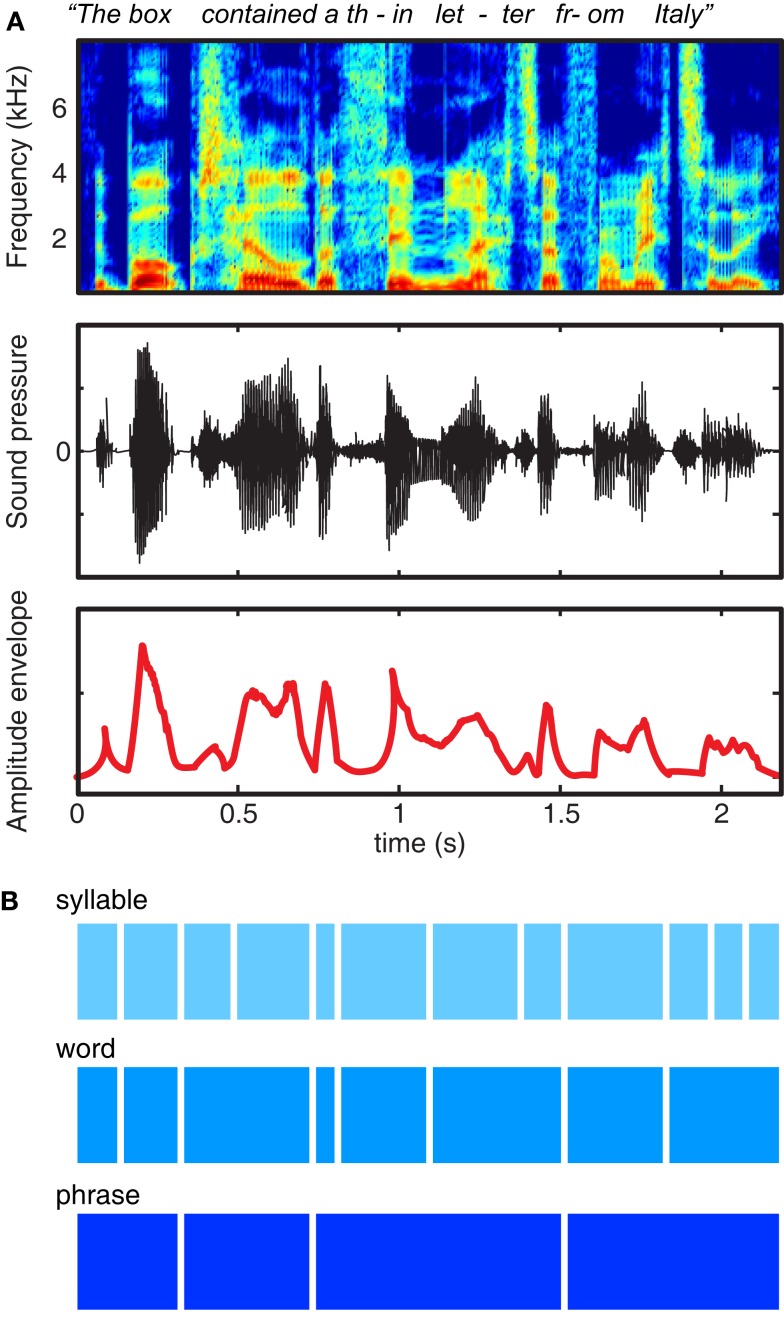
**Multiple representations of the acoustic and linguistic information in a single spoken sentence**. **(A)** At top is a spectrogram, showing power in different frequency ranges over the course of a sentence. The middle row shows the changes in sound pressure over time, as occur at the tympanic membrane. The bottom row shows the amplitude envelope of the sentence, corresponding approximately to the syllable rate, and created by half-wave rectifying and low-pass filtering the speech signal. **(B)** Schematic illustration of three different timescales of acoustic and linguistic information contained in the sentence.

Decades of experience with written language reinforces the illusion that speech is composed of sequences of regularly spaced, invariant units grouped into discrete chunks (Morais et al., 1979). However, examination of the acoustic characteristics of the speech signal – as shown in Figure 1A – quickly dispels the notion that speech consists of sequences of segments organized into words: When we speak we produce continuous fluctuations in air pressure that show little respect for linguistic boundaries. Thus, although speech indeed contains quasi-regular structure at a range of timescales, this structure does not correspond in any simple way to the letters and words that are salient in written text, nor to the key acoustic contrasts that are used to convey meaning.

The information conveyed in speech, shown schematically in Figure 1B, reflects progressively larger units of organization (syllables, words, and phrases). These acoustic structures map indirectly and imperfectly onto linguistic units: for instance, words consist of variable numbers of syllables, and looking at acoustic information alone it may be unclear to which syllable a specific segment should be assigned (such as the /m/ of “from”).

Traditional accounts of the acoustic content of speech focus on rapid acoustic transients and spectro-temporal change caused by articulatory closure and release observed during consonants and the direction and pitch of formants that contribute to the identification of vowels. However, in this review we will focus on acoustic information conveyed by the *amplitude envelope* of the speech signal. The amplitude envelope refers to the acoustic power at a given time in a given frequency range, and unless specified refers to the acoustic power summed across all frequencies (shown in the bottom row of Figure 1A). Although envelope information is well known to convey the prosodic content of a sentence (which helps communicate, among other things, emotional valence and syntactic structure), the present paper will focus on the contribution that these low-frequency fluctuations make to the identification of the linguistic content of speech: that is, identifying phonemes and words from their constituent sounds.

It is well established that the dominant component of the amplitude envelope of speech is found in temporal modulations in the 4–8 Hz range (Chi et al., 1999; Chandrasekaran et al., 2009; Elliott and Theunissen, 2009). Acoustic power in this frequency range arises from the cyclical opening of the jaw coupled with voicing (i.e., intervals containing periodic vibrations of the vocal chords). Because these acoustic characteristics are associated with events that occur once in every syllable during speech production (Greenberg, 1999), syllabic information dominates in the amplitude envelope of speech. We therefore focus on syllable-rate information, although we view this as being only one facet of a broader rhythmic hierarchy found in connected speech.

### Perception of speech with disrupted amplitude modulation

The fact that low-frequency oscillations are prominent features of the acoustic speech signal is not by itself important if listeners do not make use of this information during comprehension. However, there is ample evidence that listeners indeed rely a great deal on low-frequency temporal information in speech. An important source of evidence for this, which we will briefly review, comes from studies in which the speech signal has been altered to selectively either preserve or disrupt low-frequency acoustic information. This research suggests that acoustic information at the syllabic level plays an important role in successful comprehension.

Early evidence for the contribution of syllabic information comes from behavioral studies using interrupted speech, in which short segments of the speech signal are deleted at regular intervals (Miller and Licklider, 1950). Such studies typically find effects of both segment length and frequency of interruption, with maximal disruption of intelligibility occurring when the segments alternate at a rate approximating that of the syllable (~1–10 Hz; Miller and Licklider, 1950; Nelson and Jin, 2004; Wang and Humes, 2010). Ghitza and Greenberg (2009) extended this paradigm using speech that was time compressed to ~30% of its original duration before silent intervals were inserted. They found that identification of spoken sentences was optimal when the amount of silence inserted was chosen to return the speech signal to its original temporal structure (i.e., such that the syllable rate matched the original), consistent with listeners’ preference for syllable rates that approximate the rate of low-frequency information in the natural speech signal.

Additional support for the importance of low-frequency information to speech comprehension comes from looking at listeners’ perception of alternated speech, a related manipulation in which the speech signal is presented monaurally to the left or right ear in rapid alternation (Cherry and Taylor, 1954). Again, the largest decrements in speech intelligibility are found when the speech alternation rate would be maximally disruptive to syllable structure (Huggins, 1964). Supporting the hypothesis that this is due to informational content of speech (i.e., syllable structure), the maximal disruption tracks with speech rate, such that as speech is time compressed the poorest behavioral performance is found at a rate corresponding to the syllable rate of the faster speech (Wingfield and Wheale, 1975; Stewart et al., 2008).

A further manipulation that points to a key role of syllable-rate information in comprehension comes from a form of distortion in which successive short intervals from a spoken sentence are temporally reversed (Saberi and Perrott, 1999; Kiss et al., 2008). Despite this manipulation reversing the direction of rapid spectro-temporal changes in speech, intelligibility remains high even when intervals of 50 ms are reversed. Critically, the interval that leads to maximum disruption does not depend on the absolute duration of speech that is time-reversed, but rather on the relationship between interval duration and syllable rate: For speech that is synthesized at a higher-speech rate, disruption to speech intelligibility occurs with shorter reversals (Stilp et al., 2010). Hence, again, comprehension is lowest when syllable-rate intervals are disrupted, irrespective of speech rate.

**Figure 2:**
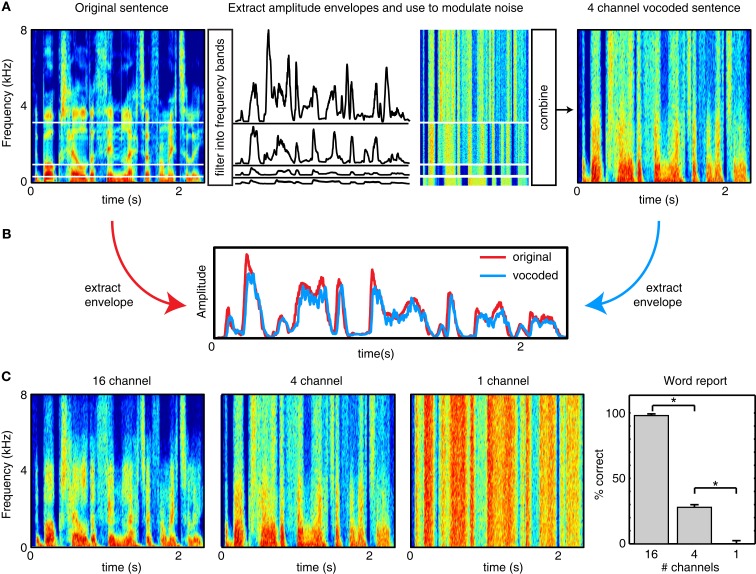
**Illustration of noise vocoding (after Shannon et al., 1995)**. **(A)** The frequency range of a stimulus is divided into a number of frequency channels (in this case, 4), usually logarithmically spaced to approximate cochlear processing. For each channel, the original sound is filtered to retain information in the given frequency range, and the amplitude modulation profile (envelope) is extracted, typically by rectification and filtering (e.g., Shannon et al., 1995) or using a Hilbert transform (e.g., Smith et al., 2002). Each amplitude envelope is used to modulate white noise filtered into the same frequency band. The amplitude-modulated white noise is then combined to form a vocoded stimulus that has significantly reduced spectral detail compared to the original speech. The more channels included in the vocoder, the more spectral detail results leading to more intelligible speech. **(B)** The overall amplitude envelope of a clear and vocoded sentence are nearly identical. Thus, although vocoded speech can differ markedly in intelligibility from clear speech, it retains the low-frequency amplitude modulations critical for perceiving speech rhythm. **(C)** Examples of the same sentence vocoded with 16 channels, 4 channels, or 1 channel. Fewer channels result in less spectral detail, as well as lower intelligibility (word report data from Peelle et al., in press).

Finally, an additional class of studies has used signal processing methods to remove (or reduce the impact of) different modulation frequencies from speech. For instance, listeners are able to understand speech dominated by low-frequency temporal cues (below ~30 Hz) provided that sufficient spectral detail is retained (Baer and Moore, 1993; Shannon et al., 1995; Elliott and Theunissen, 2009), as can be achieved with vocoded speech (see Figure 2); that is, when temporal information below 30 Hz is present in a variety of frequency bands. This is especially true if listeners are given the opportunity to train on the degraded speech (Shannon et al., 1995; Davis et al., 2005; Peelle and Wingfield, 2005). Conversely, when speech envelopes in multiple frequency bands are filtered such that information below ~16 Hz is removed, intelligibility decreases substantially for both spoken sentences and phonemes, especially consonants (Drullman et al., 1994a,b). A particularly relevant example is found in Ghitza (2012), who used a stop-band filter to flatten the modulation envelope between 2 and 9 Hz. This manipulation was found to significantly decrease intelligibility, although this effect was moderated when rhythmic information extracted from this same frequency region was re-incorporated into the final signal.

Taken together these studies provide converging evidence to demonstrate that listeners rely on information conveyed in the low-frequency amplitude modulations of the speech signal. When this information is present – and combined with sufficient spectral detail – comprehension is relatively preserved; when absent or degraded, comprehension suffers dramatically.

Before continuing, in the next section we briefly offer working definitions of three terms that pervade any discussion of low-frequency information in speech.

## Periodicity, Rhythm, and Entrainment

It is important to emphasize that amplitude modulations in naturally produced speech – as opposed to when counting, singing, or speaking in time to a metronome – are not strictly periodic, but rather quasi-periodic. That is, there are no perfectly regular acoustic cues available to listeners. This distinction is important because any discussion about oscillatory brain signals and their relationship to acoustic information necessarily raises questions about the form of periodicity found in speech. The lack of true periodicity in many complex biological systems has been a challenge for formal models of synchronization, although more recent frameworks attempt to reconcile the issue (Cummins, 2011). Operationally, speech phase has been quantified in several ways, including windowed frequency analysis approaches such as a Fast Fourier Transform (FFT). When applied to a quasi-periodic signal, this type of analysis is generally thought of as identifying the dominant phase of the signal; however, the consequences of this simplified operationalization have yet to be fully explored. More recently, Bayesian computational approaches have been developed which may prove to more accurately reflect underlying phase patterns (Turner and Sahani, 2011).

In the context of our present discussion, the critical issue is not whether the speech signal is periodic or aperiodic *per se*, but rather that it is not random: speech contains fluctuations of energy that appear at somewhat regular or predictable intervals. As we will discuss further below, these quasi-regularities can be shown experimentally to inform listeners’ expectations. Hence, although absolute regularity of timing is not necessary for speech perception, these non-random low-frequency amplitude modulations are present in connected speech and can be shown to facilitate comprehension. We will use the term *rhythm* to refer to this temporal structure – despite the fact that the speech signals we are exploring are not strictly rhythmic in the way that speech paced by a metronome would be. The key is that temporal information in speech enables listeners to make predictions about the incoming signal.

The term *entrainment* also may have a variety of interpretations. Here we use entrainment to describe the adjustment of one quasi-periodic system (neural oscillations) to match the phase of an external periodic or quasi-periodic stimulus (e.g., speech rhythm). This process is sometimes also referred to as synchronization. As expanded upon below, in neuroscience entrainment is often operationally defined as phase locking between recorded neural responses and some sort of external stimulus (Lakatos et al., 2005, 2008; Besle et al., 2011), a viewpoint that we also adopt here. Phase locking is established by demonstrating a consistent phase lag between stimuli and neural responses measured over time, over trials, or over participants. (A consistent phase of neural response to the same stimulus are sometimes interpreted in a theoretically similar matter, in that it indicates that the phase of an oscillatory brain signal is impacted by stimulus characteristics in a consistent way.)

Having clarified our use of these important terms, we now discuss in more detail the type of information provided by rhythmic information in speech.

## Functional Contribution of Speech Rhythm to Comprehension

Based on the preceding behavioral evidence, we propose that amplitude fluctuations in low-frequency ranges that are approximately the same duration as a spoken syllable (~4–8 Hz in English) are the key acoustic cue that conveys the temporal characteristics of connected speech. However, slow fluctuations alone are insufficient for comprehension: A 1 channel vocoded stimulus preserves amplitude fluctuations and speech rhythm, but is unintelligible (see Figure 2). Why then do we propose that speech rhythm plays a critical role in conveying comprehensible speech? In this section, we describe ways in which other acoustic and linguistic information conveyed by speech depends on rhythmic characteristics for accurate perception, illustrating ways in which speech rhythm makes more substantial contributions to comprehension than has previously been considered.

### Speech rhythm facilitates prediction

The slow amplitude fluctuations that we have described correspond to quasi-regularly timed events during speech production that necessarily imbue a degree of rhythmic acoustic structure onto spoken language. Speech production relies on the coordination of muscles related to breathing, lip, and tongue movement: these cyclic mouth movements (e.g., the opening and closing of the jaw) lead to rhythmic changes in speech amplitude which are associated with spoken syllables (MacNeilage, 1998). Within this structure, stressed syllables are constrained to occur at predictable times based on both linguistic attributes and the properties of the speaker’s motor system. This predictability facilitates both comprehension and inter-speaker coordination.

Consider, for example, repeatedly speaking the phrase “big for a duck.” English speakers repeating this phase tend to cluster their production into a small number of metrical structures, reflecting speakers’ systematic organization of stress patterns (Cummins and Port, 1998). Furthermore, as speech rate varies, the stress patterns change in predictable ways: at a normal speaking rate speakers tend to stress “big” and “duck” (**big** for a **duck**), whereas at slower rates additional stress is added on “for” (**big for** a **duck**), but not “a.” Furthermore, when two speakers are asked to read aloud in time with one another, they are able to synchronize their speech production (Cummins, 2003), under certain circumstances even in the absence of intelligibility (Cummins, 2009). That is, even though the acoustic signature of this rhythm is not perfectly regular – as determined by measurements of the duration of linguistic elements – there is sufficient structure for coordination of acoustic events between speaker and listener to occur. This is consistent with talkers and listeners being sensitive to a hierarchical rhythmic structure in speech.

The natural tendency of speakers and listeners to entrain to a common rhythmic structure that links elements of articulatory mechanics (jaw aperture) and of auditory perception (amplitude envelope) provides a scaffold on top of which other temporal characteristics of speech can be organized. Below we offer two examples of this, at both a narrower and a broader timescale than the syllable. We first review evidence that the temporal structure of a syllable-initial stop and the onset of voicing are perceived in relation to the timing of amplitude modulations in the preceding acoustic signal. We then provide examples of how the perception of larger lexical and syntactic phrases is guided by expectations based on the preceding speech rate. Thus, for both sub-syllable units (stops) and supra-syllable units (words or phrases) we suggest that perceptual processing is best explained by considering the timing of these elements within a rhythmic hierarchy organized around quasi-regular, syllabic intervals. These intervals provide listeners with a reference frame that can be used to distinguish the timing of critical acoustic cues in speech.

### Syllable rhythm influences perception of sub-syllabic elements in speech

Our first example comes from considering the difference between syllable-initial unvoiced and voiced stops like /p/ and /b/. A multiplicity of acoustic-phonetic cues serve to distinguish syllables like /pa/ and /ba/ in English. However, perhaps the most robust of these cues is a change in the relative timing of the release of articulatory closure and the onset of vocal chord vibration, or voice onset time (VOT; Lisker and Abramson, 1964). Although VOT is a continuous dimension, listeners perceive syllables categorically: that is, in everyday speech, listeners hear intermediate acoustic tokens as being either a /pa/ or a /ba/, as opposed to some undefined phoneme. Thus, by holding other acoustic cues constant and manipulating VOT, it is possible to plot a categorical perception curve indicating the VOT at which a listener’s perception changes from /pa/ to /ba/ (Liberman et al., 1961).

The perception of phonemic contrasts such as between /pa/ and /ba/ does not occur in isolation, but can only be explained by consideration of the complex acoustic, articulatory, lexical, and phonetic context in which they occur (Ganong, 1980; Summerfield, 1981; Holt, 2005). Most strikingly for our purposes, the rate of ongoing speech alters listeners’ phonetic categories, such that the same acoustic sound can be perceived as different phonemes depending on the surrounding speech rate (Port, 1979; Miller, 1981; Summerfield, 1981; Miller et al., 1984). In other words, cues like VOT are not objective, but processed relative to surrounding speech content. Within our account, speech rhythm (and hence speech rate) is calibrated by reference to ongoing slow amplitude modulations dominated by stressed syllables. Because these stressed syllables coincide with speech rate, the amplitude envelope also conveys information about speaking rate. We therefore propose that acoustic amplitude modulations support the necessary rate dependency in categorizing syllable initial stops as voiced or unvoiced. In a later section, we expand this proposal and illustrate how oscillatory neural mechanisms that entrain to ongoing syllables provide a natural account of the way changes in VOT categorization arise as a function of ongoing speech rate.

### Syllable rhythm influences perception of supra-syllabic elements in speech

**Figure 3:**
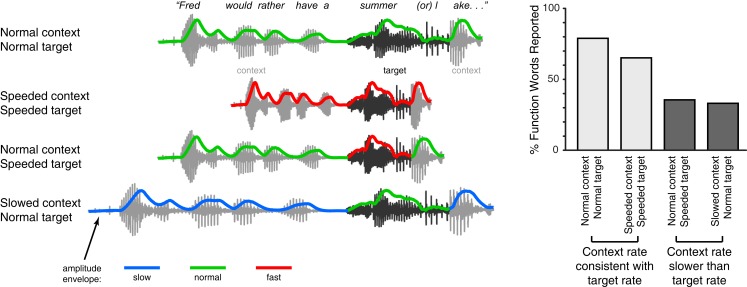
**Schematic illustration of experimental manipulation and findings of Experiment 1 in Dilley and Pitt (2010)**. Listeners heard a sentence containing an acoustic token of a function word with minimal cues to word boundaries due to co-articulation. For example, in the sentence fragment “Fred would rather have a summer or lake…,” the word “or” is present, but underarticulated. The speech rate of either the target fragment (“summer or l-”) or its context was then selectively manipulated, and the effect of these manipulations on listeners reporting the function word (in this example, “or”) was measured. These conditions are shown along the left side of the figure, along with the acoustic amplitude envelope for each stimulus (colored lines). The authors found that when the speech rate of the context and target word matched, word report for the function word was relatively high; by contrast, when the context was slower than the target word, fewer function words were reported. This result shows how listeners make use of contextual speech rate to guide lexical segmentation during speech comprehension.

In addition to phonemes, effects of speech rate are also seen in the perception of larger linguistic units like words and phrases. For instance, transient lexical ambiguities in the perception of syllables like “cap” (which may be an isolated word or the start of a longer word like “captain”) are resolved in part using acoustic cues such as syllable duration (Davis et al., 2002; Salverda et al., 2003). Interestingly, the timing information that is used to distinguish such syllables need not be local to the ambiguous syllable: Speech-contingent eye-movements show that manipulations of the timing or prosodic characteristics of the preceding sentence can modulate perception of a lexically ambiguous syllable such as “pan” vs. “panda”(Salverda et al., 2007; Brown et al., 2011). A similar effect is seen for more ambiguous acoustic tokens such as the sequence “note book worm,” which depending on prosodic information can be heard as “notebook worm” or “note bookworm.” The way in which listeners parse such an ambiguous acoustic token is significantly impacted by duration cues in prior segments (Dilley and McAuley, 2008), even if the preceding speech is low-passed filtered to remove lexical content (Dilley et al., 2010). Furthermore, the perception of unstressed syllables is drastically affected by the speech rate heard in preceding speech. As shown in Figure 3, artificially making the speech rate in the preceding sentence slower than that of a target phrase can lead listeners to miss an acoustically present word (e.g., hearing “summer lake” instead of “summer or lake”); conversely if the preceding rate is faster than that of the target phrase then listeners may report hearing an (acoustically absent) function word (Dilley and Pitt, 2010). These results suggest that listeners’ perception is significantly influenced by contextual speech rate, which in turn affects the interpretation of acoustic cues. A straightforward hypothesis coming out of this observation is that pauses reflecting higher-level narrative and syntactic structure (Gee and Grosjean, 1984; Swerts and Geluykens, 1994) would also be interpreted relative to contextual speech rate. Thus at lexical and higher levels, rate-based processes play a crucial role in guiding comprehension.

Given the dominant contribution of low-frequency information to the speech signal, and listeners’ reliance on the information it conveys at multiple levels of analysis, a natural next step is to ask how the human brain might process this information during natural language comprehension. Before we can discuss the processing of temporally regular intervals in speech we will first give a general overview of how oscillatory neural mechanisms contribute to the processing of sensory events more generally.

## Phase-Locked Cortical Oscillations as Mechanisms of Efficient Sensory Prediction

**Figure 4:**
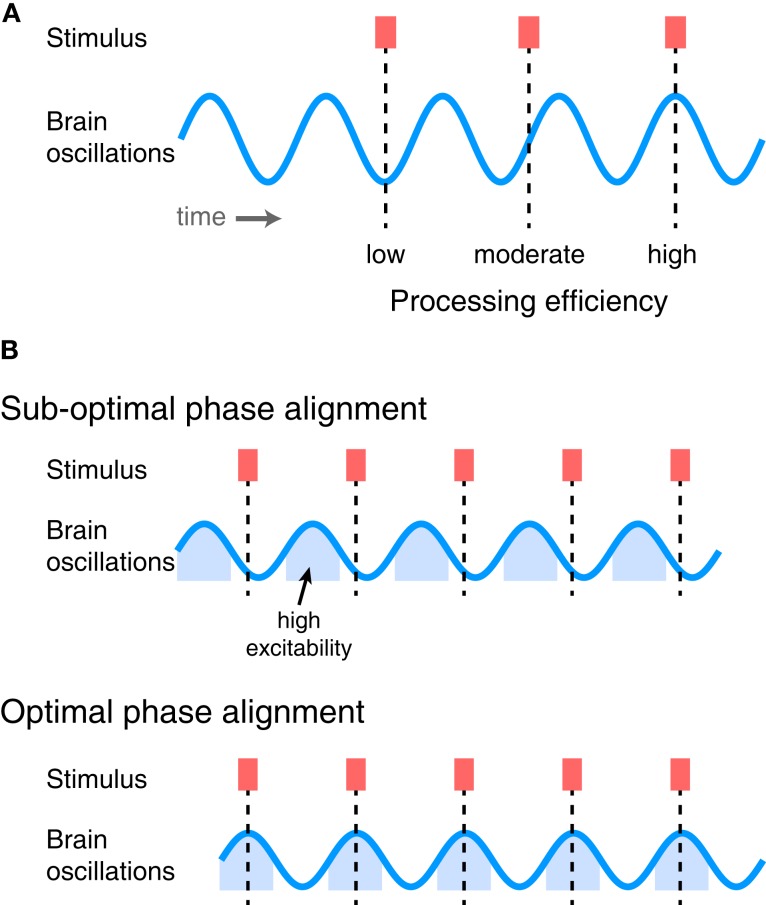
**(A)** Ongoing oscillatory activity determines how efficiently sensory stimuli drive perceptual processes, depending on the phase of oscillation at which they arrive. Information arriving at a low-excitability phase is processed relatively less efficiently, whereas that arriving at a high-excitability phase is processed more efficiently. **(B)** If sensory information exhibits temporal regularity, overall processing efficiency can be increased by shifting the phase of ongoing neural oscillations to line up with the phase of the stimuli. Top: Repeated stimuli arriving at sub-optimal phases of neural oscillations. Bottom: By shifting the phase of the brain oscillations, stimuli now arrive at a phase during which neurons are in a relatively excitable state and are thus processed more efficiently (i.e., lead to greater numbers of informative spikes).

Ensembles of neurons show systematic, synchronous fluctuations in transmembrane currents that are evident in both local field potentials measured from electrodes placed in the brain and in surface recordings measured with EEG and MEG (Bishop, 1932; Mitzdorf, 1985). These oscillations reflect shifting excitability of neuronal populations between relatively depolarized and relatively hyperpolarized states (Buzsáki and Draguhn, 2004; Lakatos et al., 2005). Given the varying excitability of neuronal populations, it stands to reason that there are some phases of oscillatory activity at which activity is closer to threshold than others (Womelsdorf et al., 2006), and that information arriving at these times will be therefore processed more efficiently, as illustrated in Figure 4. These observations have led to the view that structured oscillatory neural activity plays a crucial role in sensory prediction, processing, and attentional selection (Engel et al., 2001; Fries, 2005; Lakatos et al., 2005; Womelsdorf et al., 2007; Schroeder and Lakatos, 2009; Canolty and Knight, 2010; Schroeder et al., 2010; Arnal and Giraud, 2012; Golumbic et al., 2012).

Evidence that oscillatory neural activity contributes to perception has come from studying the relationship between sensation and spontaneous oscillations in sensory cortex. When human participants are presented with brief flashes of light at an intensity such that only approximately half of the flashes are detected, the phase distribution of spontaneous oscillatory activity (in the 6–10 Hz, alpha range) immediately prior to stimulus presentation predicts whether participants will see a particular target or not (Busch et al., 2009). This same effect has also been induced by using transcranial magnetic stimulation (TMS) to induce cortical oscillations at various frequencies, once more resulting in significant effects on participants’ perceptual detection when stimulation occurred in the ~10 Hz range (Romei et al., 2010).

Thus, if information in the environment – or the input to a particular neural system – occurs with a regular temporal structure, it can be processed with maximum efficiency if neural oscillations in the receiving region are aligned such that inputs arrive at a time of high excitability, as illustrated in Figure 4B. Oscillations that are phase-locked to rhythmic stimuli can therefore be thought of as making a prediction, or encoding a neural expectation, about when critical information is likely to arrive (Engel et al., 2001). By ensuring that relevant inputs arrive at periods of high neural excitability, there will be the optimal opportunity for this information to be processed quickly and efficiently and drive perception and behavior. In order to maintain optimal sensitivity, however, ongoing neural oscillations must be realigned in order to match the expected occurrence of sensory input. This necessitates adaptive processes that adjust the timing of oscillatory neural activity to fit the perceptual input and behavioral goals of the organism.

This alignment of ongoing oscillatory activity to rhythmic stimuli has been observed in recordings of neural activity in sensory cortex under different attentional conditions (Lakatos et al., 2008; Schroeder and Lakatos, 2009). Multiunit and local field potential recordings in monkey cortex show that low-frequency (delta) oscillations entrain to rhythmic stimuli in an attention-dependent manner (i.e., entrainment depends on attention). Furthermore, the phase of these oscillations is systematically related to both (a) response magnitude (with bigger responses occurring during high-excitability phase of delta oscillations) and (b) reaction times (with faster behavioral responses occurring to stimuli during the high-excitability phase of delta oscillations; Lakatos et al., 2008). Similar attention-related effects on phase locking to rhythmic stimuli have also been reported in human auditory cortex using electrocorticographic (ECoG) techniques (Gomez-Ramirez et al., 2011).

As we have seen, the phase locking of low-frequency oscillations to rhythmic stimuli is a key neural mechanism by which an organism can be optimally sensitive to relevant perceptual inputs. One process that allows the phase of low-frequency oscillation to exert these effects is the modulatory effect that low-frequency oscillations have on higher-frequency neural activity. For example, in humans the phase of low-frequency (in this case, theta) oscillations has been shown to modulate power in higher-frequency (gamma) oscillations in a variety of tasks (Canolty et al., 2006; Belluscio et al., 2012). These nested oscillations are a dominant feature of cortical processing both for spontaneous activity and for activity during ongoing cognitive tasks (He et al., 2010). Furthermore, the hierarchical nature of cross-frequency coupling (in this case, theta-to-gamma) appears to be a fundamental principle underlying neural communication and coordination (Fries, 2005; Jensen and Colgin, 2007; Womelsdorf et al., 2007; Canolty and Knight, 2010). Thus, the effects of low-frequency phase locking in auditory and visual cortex are likely to impact on a distributed set of neural computations at different frequencies and in different regions. With respect to acoustic processing, this type of nested hierarchical organization may help optimize the neural response to stimuli across multiple timescales (Lakatos et al., 2005; Giraud and Poeppel, 2012).

## Neural Entrainment to Speech Rhythm

Having seen the important role played by neural oscillations that entrain to regular visual or auditory stimuli, it is natural to ask whether the brain responses to speech show a similar form of entrainment. This seems likely based both on the inherent quasi-regular structure of speech, and behavioral evidence reviewed earlier that preservation of this rhythmic structure is key to successful speech perception and comprehension. Consistent with these observations, the past decade has seen an increasing number of studies providing support for the hypothesis that neural oscillations track the low-frequency amplitude envelope of the speech signal, and in turn support computational accounts in which amplitude envelope drives perception of syllabic units in speech (Hartley, 2002). Our definition of “tracking” here is not that neural oscillations provide an absolute copy of the amplitude envelope – although such representations likely exist at peripheral levels of the auditory system – but that the phase of ongoing oscillations is adjusted to more closely match the dominant phase of the (quasi-regular) amplitude envelope of speech. In other words, there is a systematic relationship between the phase of the neural signal and the phase of the amplitude envelope that arises from a tendency of neural systems to adjust and adapt to the rate and timing of quasi-regular signals in speech.

An early study that showed a link between oscillatory neural responses and processing of the speech envelope was reported by Ahissar et al. (2001). The authors presented listeners with a small set of short sentences with similar amplitude envelopes (e.g., “black cars can all park”/“black dogs cannot bark”). These sentences were time compressed to between 20% and 75% of their original duration as a way to manipulate speech rhythm and intelligibility in parallel (see also Nourski et al., 2009; Hertrich et al., 2012). The authors found that when the speech was intelligible, MEG global field power showed a peak in the frequency spectrum that matched the dominant modulation frequency in the amplitude envelope of the sentences (around 5 Hz). However, for severely time-compressed sentences that contained higher-frequency modulations (10 Hz or greater), this close relationship between modulation frequencies in the speech signal and the MEG responses disappeared. Compellingly, individual differences in behavioral accuracy on a task that is contingent on intelligibility (true/false judgment) were predicted by how closely matched neural signals in individual listeners were to the acoustic envelope of the speech signal. These data thus provide evidence relating cortical oscillations to low-frequency modulations in the speech amplitude envelope, and furthermore suggest that these cortical responses may be related to the intelligibility of the speech. However, this study left the relationship between speech intelligibility and neural entrainment unresolved: it could be that the brain’s inability to track the speech signal resulted in the speech being unintelligible, but the opposite is equally possible (i.e., that when speech is unintelligible, neural oscillations do not track it well). We return to the issue of speech intelligibility below, but for now emphasize the evidence for a systematic relationship between the acoustic speech envelope and cortical oscillations.

A significant step forward in this line of inquiry was achieved by Luo and Poeppel (2007), who used MEG to examine the ability of cortical oscillations to distinguish between individual sentences based on their acoustic information (see also Suppes et al., 1997; Suppes et al., 1998, 1999). Luo and Poeppel found that phase information in the 4–8 Hz range, but not at other frequencies, was able to accurately discriminate between intelligible sentences, though this was reduced for unintelligible versions of the same sentence. Notably, it was not possible to distinguish between intelligible sentences using the power spectrum of the MEG response, only phase information. Moreover, analysis of pre-stimulus activity suggested that ongoing oscillations modulated their phase to match the acoustic information in the speech signal. Because the low-frequency modulations in the acoustic signal differed between stimuli, phase-locked responses to this input were able to accurately distinguish between sentences.

An elegant extension of this finding was reported by Kerlin et al. (2010) using EEG, in which listeners simultaneously heard two different sentences, one to each ear, along with visual instructions indicating the side to which they should attend. The authors first constructed a group-averaged waveform, reflecting the average neural response to a sentence across all listeners. They found that selective attention resulted in the EEG signal of an individual listener showing a better match to the group response to the attended sentence, most notably in the 4–8 Hz range. There are two key points made by this study. First, the phase-locked response to a sentence is relatively consistent over individuals, as evidenced by the neural response by an individual listener showing correspondence with the group average. This suggests that different listeners’ brains are entraining to a common acoustic cue in the speech signal. Second, the acoustic information was matched in the attended and unattended conditions: If acoustic cues were all that mattered, the neural response should also be identical across these two conditions. Thus, the enhancement of the ongoing response to speech by selective attention suggests that listeners’ phase-locked response is not only responding to acoustic information. This finding is consistent with work in other domains demonstrating similar attention-related effects on the phase of cortical responses (Lakatos et al., 2008; Gomez-Ramirez et al., 2011), as well as with studies that use a linear-system identification method to assess the neural response associated with speech envelope signals (Lalor and Foxe, 2010) which differ in attended and unattended conditions (Power et al., 2011).

### Phase resetting as a mechanism of neural entrainment to speech

**Figure 5:**
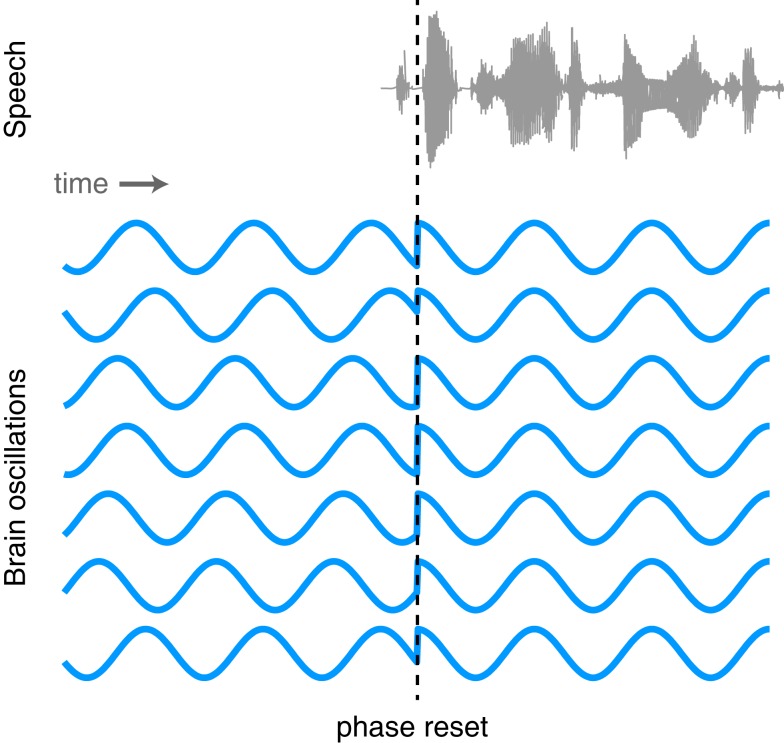
**Illustration of the phase of ongoing neural oscillations being reset by an external stimulus**. Prior to the stimulus event the phases of the oscillations are random, but following the stimulus they are aligned.

The proposed link between neural oscillations and the speech signal naturally leads us to consider what functional mechanism is responsible for achieving such neural entrainment. One possibility is that the phase of neural oscillations is reset by stimulus onsets. Such phase resetting is a common phenomenon observed at multiple levels of systems neuroscience (Makeig et al., 2002; Rizzuto et al., 2003; Lakatos et al., 2005; Burgess et al., 2007). As illustrated in Figure 5, oscillations that exhibit a random phase before the onset of stimulus take on a consistent phase following the onset of sensory input. The event triggering the phase reset might be a salient sensory stimulus (i.e., the beginning of a sound), a word that will later need to be recalled, or any one of a number of identifiable neural phenomena.

Although evidence for phase resetting in response to speech signals is limited, as noted above Luo and Poeppel (2007) have reported that the phase (but not amplitude) of neural oscillations in the 4–8 Hz range was able to discriminate between spoken sentences. This is consistent with the proposal that speech signal lead to phase resetting although it is unclear whether this resetting necessarily occurs at or soon after the onset of an utterance as shown in Figure 5, or may in fact be sensitive to various peaks in the amplitude envelope. As we noted previously, the growing body of work supporting phase locking between neural oscillations and the acoustic speech signal is consistent with phase resetting, though other mechanisms (e.g., more gradual phase-shifts) have not been ruled out. Thus, phase resetting seems a likely candidate for a mechanism of oscillatory entrainment of brain signals and speech acoustics (Ghitza, 2011; Giraud and Poeppel, 2012) and further neural and behavioral evidence for the mechanisms involved would be valuable.

## Do Phase-Locked Responses to Speech Depend on Intelligibility?

A matter of ongoing debate centers on whether phase-locked responses to speech reflect any degree of linguistic processing (i.e., comprehension), or are merely responses to the acoustic information in speech. It is certainly the case that phase-locked responses to sensory stimuli are not unique to human speech comprehension, as evidenced by oscillatory responses to simple auditory stimuli in non-human primates (Lakatos et al., 2007, 2008). Might there be additional information in the speech signal that affects the phase-locked cortical response to spoken language in humans?

As noted above, several studies suggest this possibility by reporting an association between the intelligibility of time-compressed speech and the degree of phase locking seen in cortical oscillations (Ahissar et al., 2001; Nourski et al., 2009; Hertrich et al., 2012). However, the causal nature of this relationship was not able to be determined based on these data: Although one interpretation might be that as speech became less intelligible the phase-locked responses to the acoustic signal were reduced, an equally plausible reading (and the one supported by the authors) is that as the brain became less able to track the acoustics of the speech signal, intelligibility suffered. Under this view, the correlation between cortical responses phase-locked to speech and intelligibility reflects the ability of the brain to process acoustic information, and not linguistic information.

Subsequent research appears to support this interpretation. Luo and Poeppel (2007) manipulated speech intelligibility using speech-noise chimeras (Smith et al., 2002), and found that as intelligibility decreased, the ability of phase information to discriminate between different sentences was reduced. Howard and Poeppel (2010) explicitly focused on the issue of speech intelligibility by presenting listeners with normal and time-reversed sentences (which are unintelligible), and found that phase information could discriminate between the time-reversed sentences with an accuracy comparable to that of the normal sentences. They conclude that oscillations show phase locking to acoustic cues that tend to be associated with speech intelligibility (e.g., acoustic transients), but do not depend on intelligibility itself.

However, there remain reasons to question this conclusion. In a recent MEG study, Peelle et al. (in press) presented listeners with noise-vocoded sentences that varied in intelligibility. They found significant bilateral phase-locked responses to unintelligible, 1 channel vocoded speech. Such responses would be able to distinguish speech stimuli based on tracking the acoustic amplitude envelope in the absence of linguistic content, and could therefore explain the high discriminability that is able to be achieved between unintelligible stimuli (Howard and Poeppel, 2010). When speech was intelligible, these phase-locked responses were enhanced in the left hemisphere. These results suggest that oscillatory neural responses to speech include some component of general sensory processing that occurs regardless of intelligibility, but are enhanced in the left hemisphere by the presence of linguistic information available in intelligible speech.

An additional consideration involves the specific manner in which intelligibility is manipulated. Above, we have made the argument that the low-frequency information in the acoustic envelope, corresponding approximately to the syllable rate in spoken language, plays a critical role in speech comprehension. Table 1 lists all of the MEG, EEG, or ECoG studies of which we are aware that have investigated phase-locked responses to connected speech that varies in intelligibility. Of special note is that in four cases, the intelligibility manipulation also alters the amplitude envelope, making the relationship between speech acoustics, comprehension, and phase-locked oscillatory responses unclear. In the cases where the speech envelope was preserved (Luo and Poeppel, 2007; Peelle et al., in press), the concomitant reductions in intelligibility and phase-locked oscillatory responses are consistent with a systematic relationship between these two factors.

**Table 1:** **Studies examining the effect of intelligibility on the phase locking of cortical oscillations to the speech signal**.

Study	# Sentences	Intelligibility manipulation	Amplitude envelope preserved?	Phase-locked responses to less intelligible speech
Ahissar et al. (2001)	18	Time compression	No	Decreased
Luo and Poeppel (2007)	3	Speech-noise chimera	Yes	Decreased
Nourski et al. (2009)	10	Time compression	No	Decreased^a^
Howard and Poeppel (2010)	6	Time-reversed speech	No^b^	Equivalent
Hertrich et al. (2012)	40	Time compression^c^	No	Decreased
Peelle et al. (in press)	200	Noise vocoding	Yes	Decreased

*^a^The authors report a lack of phase-locked responses at speech rates where intelligibility suffered; however, they also report amplitude-related fluctuations in power at higher-frequency regions, which suggests at least some sort of neural following of the amplitude envelope even at these extremely fast speech rates*.

*^b^The temporal reversal results in a non-equivalent amplitude envelope for the reversed sentences, but one that shares many of the overall properties, such as power in the low-frequency spectrum and amplitude profiles during relatively steady-state portions of the speech signal (e.g., at the middle of a vowel or consonant closure). However, many acoustic transients have a characteristically asymmetric temporal profile that will be disrupted by time reversal and have significant perceptual consequences (e.g., Irino and Patterson, 1996)*.

*^c^ Intelligibility data for these stimuli is reported in Hertrich et al. (2009) and show a substantial decline in word report for time-compressed speech presented at ~16 syllables/second (greater than 50% compression)*.

Thus, on balance the majority of the evidence suggests that linguistic content in speech affects the prediction of acoustic cues, leading to an increase in phase-locked neural oscillations during speech comprehension.

## Functional Contributions of Neural Entrainment to Speech Perception: Perception of Voicing Contrasts as an Example Case

**Figure 6:**
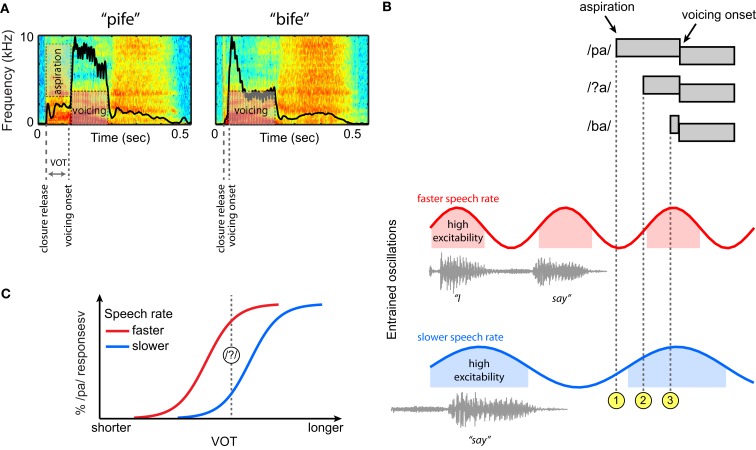
**Hypothesized contribution of entrained neural oscillations to categorical phoneme judgments based on voice onset time (VOT) in connected speech**. **(A)** Spectrograms of two non-words, “pife” and “bife.” The amplitude envelope for each is overlaid in black. **(B)** At top, schematic illustrations of three phonetic tokens (/pa/, /ba/, and an ambiguous intermediate token, /?a/) that differ in VOT. Neural oscillations entrained to two different speech rates are shown below, here for the short carrier phrase “I say̲̲̲.” For both speech rates, the aspiration for a clear /pa/ occurs in a region of low excitability of the entrained oscillation (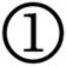), and the aspiration for a clear /ba/ in a region of high excitability (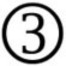). However, for the ambiguous token, the aspiration occurs at different levels of excitability for the faster and slower speech rates (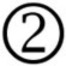), making it less likely to be perceived as /pa/ (and more likely to be perceived as a /ba/) at slower speech rates. **(C)** Schematic categorical perception curves demonstrating a shift of perceptual boundaries as a function of speech rate based on this framework.

Having seen that neural oscillations entrain to low-frequency acoustic information in the speech signal, we now return to the perception of VOT differences in syllable-initial stop consonants. For this example speech contrast we provide a concrete, mechanistic illustration of how oscillatory neural mechanisms that entrain to the amplitude envelope of ongoing speech might serve to guide perception. A depiction of the relevant acoustic cues to distinguish voiced and unvoiced stops, and how these cues might be processed by an oscillatory neural system, is outlined in Figure 6.

Voice onset time provides one of the primary acoustic cues to distinguish /p/ and /b/, shown in Figure 6A in the context of two non-words (VOT being longer for /p/ than for /b/). For a syllable-initial unvoiced stop, the delay between the consonantal release and the onset of voicing is typically filled with aspiration noise. The presence of higher acoustic amplitudes during voicing than during aspiration leads to a marked difference in the overall amplitude envelope of these two syllables. As noted previously, speech rate effects on phoneme perception are robust, such that the same physical VOT can be treated as evidence for either a voiced (/b/) or an unvoiced (/p/) stop depending on the preceding speech rate (Port, 1979; Miller, 1981; Summerfield, 1981; Miller et al., 1984, 1997). Thus, the specific question that we can ask regarding perception of these syllable initial stops is what underlying neural mechanism will allow speech-rate-contingent shifts in VOT values that cue the categorical distinction between /p/ and /b/.

Given the evidence outlined previously, we assume that neural oscillations in the brain entrain to ongoing speech rate as conveyed by the amplitude envelope. Figure 6B displays schematic representations of three acoustic tokens that differ in VOT: unambiguous tokens of /pa/ and /ba/, and an intermediate ambiguous segment /?a/ that is perceived as either /pa/ or /ba/ depending on the ongoing speech rate. Below these tokens are oscillations that have been entrained to different frequencies by preceding speech signals – that is, the speech tokens do not occur in isolation, but as part of a connected speech stream that sets rhythmic expectations for speech rate. As noted previously, the entrained neural oscillations can be thought of as encoding the predictions of the auditory system about when the onset of the next stressed syllable will occur (cued by an increase in the amplitude envelope).

In this example, the aspiration onset for a clear /pa/ always occurs in the low-excitability phase of the entrained oscillations (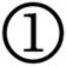), and the aspiration onset for a clear /ba/ consistently occurs in a high-excitability phase (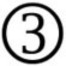). By contrast, for the ambiguous token /?a/, the aspiration onset occurs in either a low or high-excitability part of the ongoing oscillation, depending on the speech rate (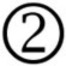). That is, with oscillations at slower frequencies – reflecting entrainment to slower speech rate – the relationship of voicing onset to the phase of the oscillation shifts. Thus, at a faster speech rate (in red), the aspiration onset for /?a/ occurs in a low-excitability portion, making it more similar to a clear /pa/. Conversely, at a slower speech rate (in blue), the aspiration onset for /?a/ occurs at a time of high excitability, and thus is more similar to a clear /ba/. This type of categorical boundary shift, illustrated in Figure 6C, is precisely the behavioral change observed when speech rate is manipulated in categorical perception studies (Miller, 1981; Summerfield, 1981).

Note that we are not proposing that any *particular* phase relationship of the acoustic signal and ongoing cortical oscillations is necessary for these speech rate effects to occur; rather, it is only the consistency of a phase relationship (i.e., its non-randomness, thus enabling prediction) that is important. Indeed, the acoustic-neural phase relationship might differ across individuals for any number of underlying neurobiological reasons, but the reinforced learning of a relationship within an individual would still make this relationship useful during connected speech comprehension.

Thus, entrained neural oscillations provide a way for the auditory system to encode ongoing speech rate and adjust perceptual boundaries for speech accordingly. Here we have focused on an illustration of how neural entrainment to syllable-rate information can alter the processing of sub-syllabic consonants. The behavioral evidence that we reviewed earlier suggests similar neural mechanisms operate in making use of temporal cues during the perception of words and phrases in connected speech.

## How Speech Rhythm Contributes to a Hierarchical Neural Model for Speech Comprehension

In the final section of the paper, we present a preliminary account of where speech rhythm is processed in the brain. In this, we build on what is known of the extended network of brain regions that support speech comprehension. These exhibit a hierarchical organization: regions of the temporal lobe near primary auditory cortex are sensitive to acoustic features of speech, whereas regions that are further removed (such as anterior temporal cortex and left inferior frontal gyrus) are relatively insensitive to these features (Davis and Johnsrude, 2003; Hickok and Poeppel, 2007; Rauschecker and Scott, 2009). An open question is how the neural mechanisms responsible for processing rhythmic information in speech fit into this hierarchy. We focus on the regions involved in processing amplitude modulations in the acoustic signal, relying primarily on data from fMRI and PET due to their anatomical specificity.

### The neural response to unintelligible and intelligible amplitude modulations

**Figure 7:**
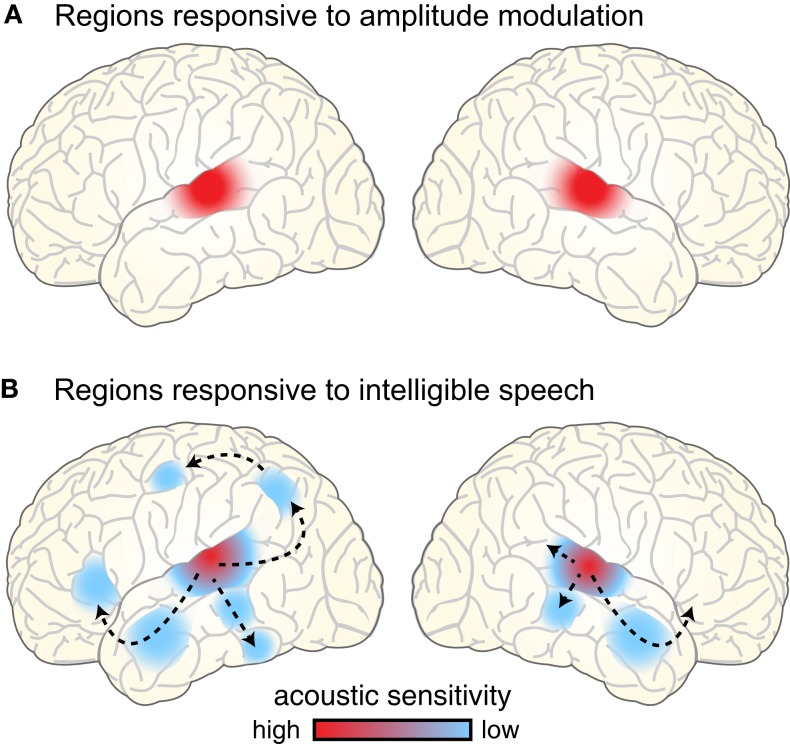
**(A)** Brain regions responding to amplitude-modulated acoustic stimulation. **(B)** Brain regions responding to intelligible speech > temporally complex control conditions. Intelligible speech – i.e., amplitude-modulated acoustic stimulation that conveys linguistic information – recruits a broad network of bilateral cortical regions that are organized into parallel hierarchies. Within this network, regions show differential sensitivity to the surface features of speech (i.e., acoustic information), with areas removed from primary auditory cortex primarily responding to the degree of linguistic information in the speech (Davis and Johnsrude, 2003).

Perhaps not surprisingly, the brain regions showing sensitivity to amplitude-modulated acoustic signals are centered near primary auditory cortex bilaterally, extending into nearby regions of superior temporal gyrus (STG; Giraud et al., 2000; Hart et al., 2003; Boemio et al., 2005; Overath et al., in press), illustrated schematically in Figure 7A. This bilateral response to amplitude modulation fits well with the presumed anatomical arrangement of phase-locked responses to speech, which are generally reported to be bilateral and responsive to unintelligible speech conditions (i.e., amplitude modulations; Luo and Poeppel, 2007; Howard and Poeppel, 2010; Peelle et al., in press). That being said, there is some evidence for hemispheric asymmetry in the preferred modulation frequency of auditory cortex, with left auditory cortex showing higher responses for rapid (~20–40 Hz) modulations and right auditory cortex for slower (~3–5 Hz) modulations (Belin et al., 1998; Boemio et al., 2005; Obleser et al., 2008). It has been suggested that these differences may reflect endogenous preferences in oscillation rate between left and right auditory cortex (Giraud et al., 2007), and support the lateralized integration of acoustic information along multiple timescales (Poeppel, 2003; Morillon et al., 2010; Giraud and Poeppel, 2012; Luo and Poeppel, 2012). However, the relationship between asymmetric processing of amplitude modulation frequencies in acoustic signals and natural speech comprehension is still not fully resolved. Indeed, imaging experiments provide clearer evidence for changes in the lateralization of neural responses caused by the outcome of speech processing (i.e., the degree of comprehension: Shtyrov et al., 2005; McGettigan et al., 2012; Peelle et al., in press), rather than due to simple acoustic characteristics of the speech signal (Obleser et al., 2008; Saoud et al., 2012). For this reason, we believe that neural regions that contribute to speech comprehension are best studied using intelligible speech rather than non-speech surrogates.

In considering the brain regions that support speech comprehension, a critical step is identifying those regions that show greater activity for amplitude modulations that convey speech information compared to amplitude modulations that do not. In other words, using amplitude-modulated stimuli (such as vocoded speech, see Figure 2) to assess where brain activity is increased for intelligible connected speech compared to unintelligible control conditions. Such comparisons consistently reveal greater activity for intelligible speech in regions of temporal cortex anterior, posterior, and inferior to primary auditory cortex, as well as in portions of left frontal cortex (Scott et al., 2000; Davis and Johnsrude, 2003; Narain et al., 2003; Rodd et al., 2005, 2010; Okada et al., 2010; Peelle et al., 2010a; Davis et al., 2011), as illustrated in Figure 7B. These parallel processing pathways reflect a hierarchy of cortical organization that is flexibly recruited in service of the acoustic, lexical, phonological, semantic, and integrative processing required to comprehend connected speech (Davis and Johnsrude, 2007; Hickok and Poeppel, 2007; Rauschecker and Scott, 2009; Peelle et al., 2010b).

When listeners hear amplitude-modulated acoustic stimuli, then, neural oscillations show phase locking to the acoustic cues, and the hemodynamically measured neural response is primarily confined to bilateral auditory regions. This includes both non-speech stimuli and speech that has been filtered to be unintelligible (while preserving the amplitude envelope, such as occurs with vocoding). When acoustic amplitude modulations are intelligible – that is, for intelligible speech – phase locking is enhanced, and neural activity cascades through hierarchical pathways into a broader network of regions involved in processing the linguistic content (cf. Figures 7A,B). Because many of these regions are involved in processing linguistic (as opposed to acoustic) information, this response is typically more extensive in the left hemisphere than the right.

We also note that in addition to the cortical processing hierarchy discussed above, subcortical structures have also been consistently implicated in the temporal processing of speech (Kotz and Schwartze, 2010; Schwartze et al., 2011, 2012; Stahl et al., 2011). Although we do not discuss these regions in detail, these findings are intriguing in part because they hint at the possibility of a shared biological basis for rhythm processing in speech and beat perception in music, which also relies heavily on subcortical structures (Grahn, 2009; Grahn and Rowe, in press).

### Anterior temporal cortex as a neural workspace for integrating speech information over time

Among the regions that respond to intelligible speech, lateral anterior temporal cortex has traditionally received a significant amount of attention since it was identified in an early and influential PET study conducted by Scott et al. (2000). The authors presented listeners with sentences that were clear speech, 4-channel vocoded speech (which is moderately intelligible), and the spectrally rotated versions of those two conditions. Importantly, the spectrally rotated versions maintain the overall amplitude envelope profile of the original, but are unintelligible. Scott et al. found a region of left anterior lateral temporal cortex – along the STG and superior temporal sulcus (STS) – which showed increased activity for intelligible speech relative to the unintelligible conditions, but did not distinguish between clear speech and 4-channel vocoded speech (i.e., the response showed acoustic insensitivity). Although, as noted above, subsequent studies have identified a number of additional regions that show increased activity for speech, left lateral anterior temporal cortex is reliably present in both direct contrasts (Narain et al., 2003; Okada et al., 2010; Peelle et al., 2010a; Rodd et al., 2010) and correlations with intelligibility (Davis and Johnsrude, 2003; Davis et al., 2011). An outstanding question remains as to the function of anterior temporal cortex in connected speech comprehension.

**Figure 8:**
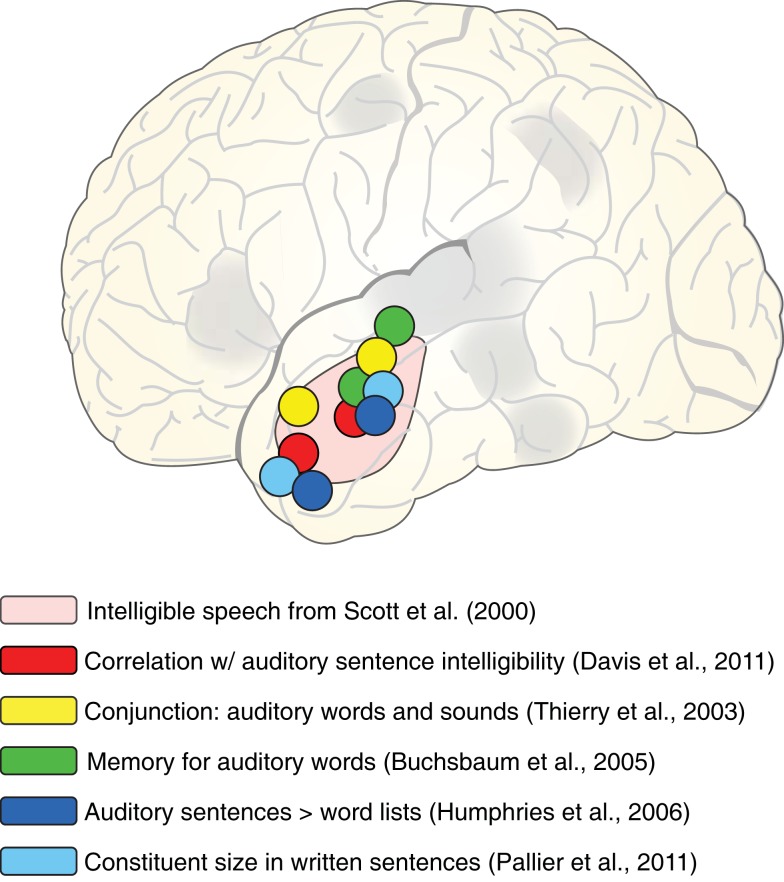
**Schematic illustration of peaks from various fMRI studies in which activation of left anterior lateral temporal cortex was observed, overlaid on the region showing sensitivity to speech intelligibility from Scott et al. (2000)**.

When considering the functional role of the anterior temporal lobe, it is helpful to consider additional conditions in which the anterior lobe is active. A sample of these tasks is shown schematically in Figure 8, overlaid on the approximate region identified by Scott et al. (2000). First, in addition to the direct intelligible > unintelligible contrast reported by Scott et al., activity in anterior temporal cortex also correlates with sentence intelligibility (Davis et al., 2011), supporting its strong relationship to the linguistic (and not just acoustic) content of sentences. This is further supported by anterior temporal cortex activity seen for listening to sentences > word lists (Humphries et al., 2006), as well as increased processing for larger constituent sizes in visually presented sentences (Pallier et al., 2011) and neural response suppression for sentences that repeat the same syntactic structure (Noppeney and Price, 2003). These findings suggest a role for anterior temporal cortex in the integration of (linguistic) meaning over time, suggested by some to support constituent building (Bemis and Pylkkänen, 2011; Brennan and Pylkkänen, 2012).

However, anterior temporal activity is not restricted to sentence-level processing. Comparable anterior temporal activity is seen for both short phrases (e.g., “car zooming by”) and matched environmental sounds (Thierry et al., 2003), as well as melody extraction (Griffiths et al., 1998), suggesting that anterior temporal activity is not specific to verbal materials. Furthermore, anterior temporal cortex plays a role in short term memory for auditory words more consistent with a shorter-lasting echoic memory than with rehearsal processes (Buchsbaum et al., 2005; Kalm et al., 2012). In both these studies, participants knew that they might be asked to recall spoken words that they heard. Thus, although the task did not require explicit semantic or syntactic integration, there was incentive to accurately encode acoustic information.

We propose that a unifying feature of these diverse tasks is that they require holding transient information in memory. Such ongoing memory is required to integrate meaningful events over time, as required by (but not unique to) connected speech comprehension. Thus, when speech is intelligible, two neural processes follow: (1) The auditory system shows greater entrainment to the ongoing amplitude modulations, and (2) linguistic information is integrated in ongoing memory supported by lateral anterior temporal cortex.

One important issue concerns the relationship between phase-locked responses to intelligible speech and increased hemodynamic responses seen in PET or fMRI studies. In a recent MEG study, we used stimuli that similar to those used by Scott et al. (2000), namely, noise-vocoded sentences that varied in intelligibility (Peelle et al., in press). As noted above, regions in the left hemisphere were identified in which phase locking was enhanced when speech was intelligible compared to the unintelligible control conditions. This suggests that when meaning can be extracted from speech, it enables left-lateralized language regions to make predictions regarding the incoming acoustic signal. Interestingly, source localization estimates suggest this enhanced phase locking is somewhat posterior – thus, closer to primary auditory cortex – than the regions reported by Scott et al. (2000). It may be that this reflects a form of top-down prediction from sentence-level processing (located more anterior in temporal cortex) to amplitude modulation processing (located closer to auditory cortex).

Together, then, low-level acoustic cues, including amplitude modulations, are processed bilaterally by auditory and peri-auditory cortex. When connected speech is intelligible, a left-hemisphere dominance emerges due to the linguistic (not acoustic) properties of the speech (Rosen et al., 2011; McGettigan et al., 2012; Peelle et al., in press). This includes an increased reliance on lateral anterior temporal cortex to support linguistic integration.

## Speech Rhythm as Part of a Nested Hierarchy of Oscillations

In this review we have intentionally focused on syllable-rate information: both the acoustic amplitude fluctuations that correspond to syllable production in the speech signal, and the neural oscillations in the range of 4–8 Hz that appear to play a critical role in processing syllables in connected speech. However, there is a wealth of information occurring at different timescales which listeners’ auditory systems also process during speech comprehension. Perhaps most obviously this includes spectral contrasts needed for segment identification, which typically occur over a much more rapid timescale than the syllable-level information emphasized here. The principles of neural oscillations and sensory processing we have reviewed would suggest that oscillations in the 4–8 Hz range would not be able to process such rapid information with maximal sensitivity. At the same time, evidence for rate sensitivity in speech perception indicates that these recognition processes at a finer timescale are informed by syllable-rate information. This naturally implies coordination of neural processes across different timescales.

The need of the auditory system to analyze multiple timescales of acoustic information in speech thus suggests multiple sampling frequencies (Poeppel, 2003), and hence multiple timescales of cortical oscillations involved in speech comprehension. Experimental and computational evidence is largely consistent with the processing of speech along multiple timescales (Ghitza, 2011, 2012; Luo and Poeppel, 2012), although evidence for the neural organization of these different timescales (e.g., the division of labor between left and right auditory cortices proposed by Poeppel, 2003) remains a topic of debate (McGettigan and Scott, 2012). As noted previously, from a neurophysiological perspective, cross-frequency coupling (e.g., the phase of neural activity in the theta frequency range modulating power in the gamma frequency range) is well-established (Lakatos et al., 2005; Canolty et al., 2006; Jensen and Colgin, 2007; Canolty and Knight, 2010). This naturally suggests at least two time windows of temporal integration for speech processing, but that the neural oscillations corresponding to these time windows are interdependent. Similar mechanisms might also operate at slower time scales suitable for processing phase or word level units depicted in Figure 1B.

Thus, acoustic amplitude modulations in the 4–8 Hz range contribute critically to the speech signal, and entrain ongoing neural oscillations during connected speech processing. However, these neural oscillations exist within a broader hierarchy of nested oscillations that support the analysis of speech along multiple concurrent timescales.

## Conclusion

Understanding spoken language requires the processing of a rapid, acoustically complex auditory signal. In the current paper we have focused on the rhythmic properties of the speech signal and the role of neural oscillations in processing this information. In closing, we emphasize the following three points:

1. Speech is inherently temporal, and low-frequency information in the range of 4–8 Hz conveyed by the amplitude envelope provides the foundation of a rhythmic hierarchy that supports the structure of spoken language.
2. Ongoing oscillations in the human brain show phase locking to low-frequency information in the speech signal, providing a mechanism to track speech rate and affect sensitivity to relevant acoustic features in connected speech. These phase-locked signals may be part of a nested hierarchy of neural oscillations that process speech at multiple timescales.
3. Amplitude-modulated acoustic signals are processed bilaterally by auditory cortex, but intelligible amplitude modulations (i.e., connected speech) rely to a greater degree on left-hemisphere regions proceeding along multiple pathways. The lateral anterior temporal lobe in particular may play a role in the ongoing memory of both spoken language and other stimuli, supporting integration processes.

The further understanding of how neural oscillations interact with speech rhythm will undoubtedly play a critical role in refining mechanistic accounts of spoken language processing.

## Conflict of Interest Statement

The authors declare that the research was conducted in the absence of any commercial or financial relationships that could be construed as a potential conflict of interest.
